# Interaction of Grafted Polymeric *N*-oxides with Charged Dyes

**DOI:** 10.1021/acs.langmuir.5c00923

**Published:** 2025-04-25

**Authors:** Erica Moretto, Michelle Kobus, Wolfgang Maison

**Affiliations:** Department of Chemistry, Universität Hamburg, Bundesstrasse 45, Hamburg 20146, Germany

## Abstract

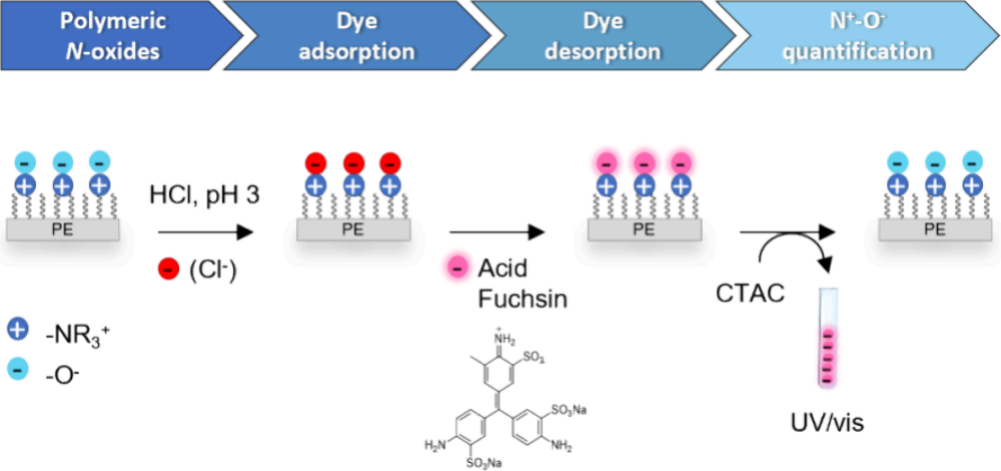

Grafted polymeric *N*-oxides have recently attracted interest for antifouling applications, drug delivery, wastewater purification, and electronic devices. Their function depends on the efficiency of the grafting process and the following postgrafting oxidation step. These two parameters govern the solvent-accessible charge density on the surface, an important parameter, which is notoriously hard to determine. In this study, a novel colorimetric quantitative assay for polymeric *N*-oxides was developed. It allows the determination of the surface charge density of grafted polymeric *N*-oxides. The method is based on the adsorption of acid fuchsin (AF) to grafted *N*-oxides through reversible electrostatic interactions between the positively charged nitrogen atoms of the *N*-oxide functionality and the sulfonate groups of the dye. The process depends thus on the pH-switchable properties of polymeric *N*-oxides. Adsorption was achieved at a pH value of 3, where *N*-oxides are almost fully protonated (typical p*K*_a_ 4–5). AF was desorbed from the surface at pH 7 and quantified via visible adsorption spectroscopy (UV–vis) at 556 nm to determine the amount of surface-grafted functional groups. Charge densities of diverse *N*-oxides grafted by free radical polymerization from polyethylene (PE) were determined to be in the range 1–3 × 10^15^ N^+^-O^–^/cm^2^. Notably, *N*-oxides can form covalent bonds with electron-deficient triarylmethane dyes like AF. This nucleophilic reactivity of *N*-oxides does not compromise the proposed assay, but it may be of relevance for dye adsorption and desorption in wastewater purification.

## Introduction

**Scheme 1:**
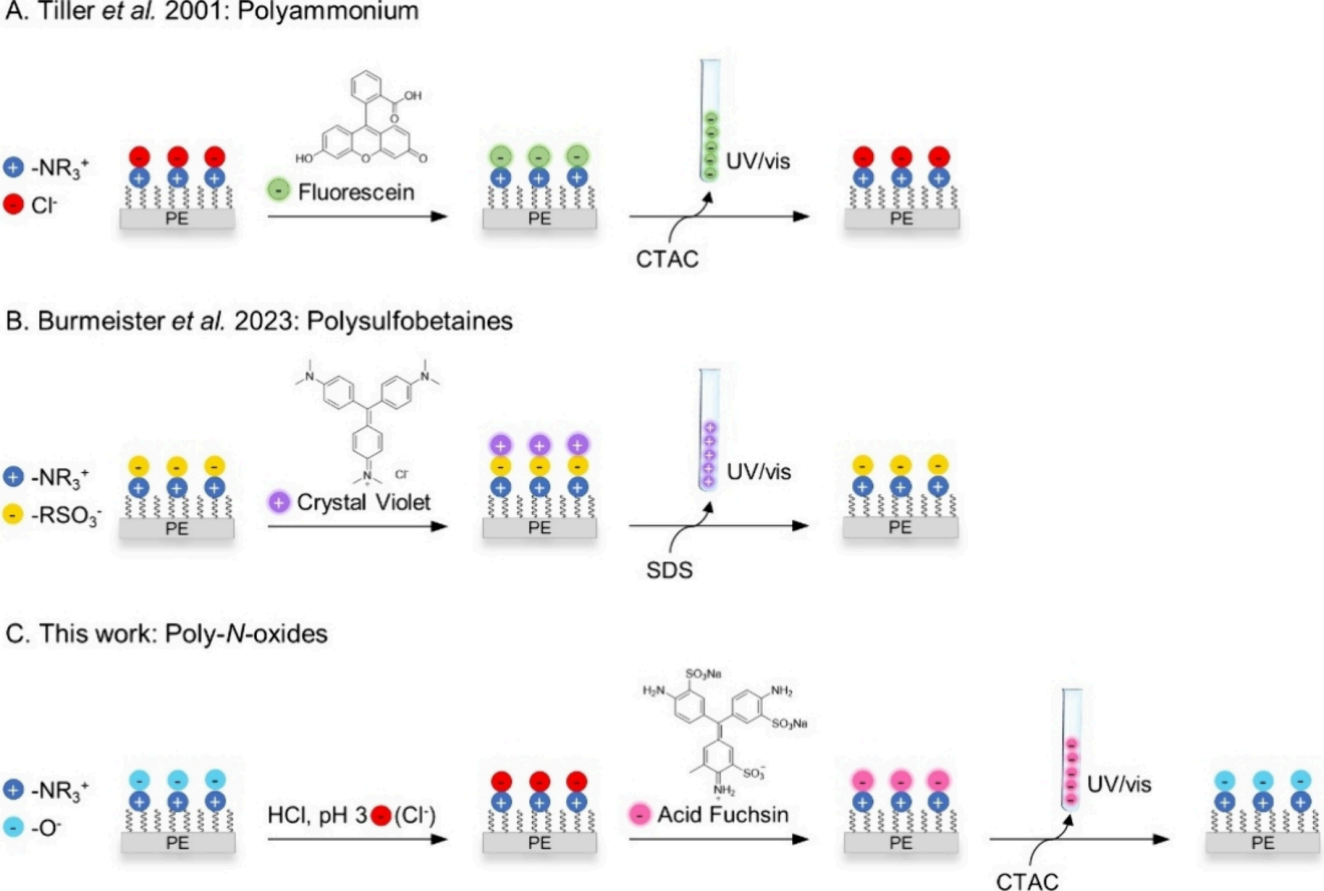
Schematic Drawing of Colorimetric Assays for the Analysis of Charged Polymer Brushes on Bulk Polyethylene

Grafting of charged polymer brushes onto surfaces is a common method to prepare new functional materials.^1^ The functional groups of the applied polymer brush layers are exposed to the environment, thus shaping important properties of the material. They can, for example, provide the material with a chemical reactivity, electric conductivity or antifouling properties.^2,3^ For example, cationic polymer brushes have contact-active antibacterial properties^4−6^ and zwitterionic polymer brushes have low-fouling properties.^7^ The latter depends on the high hydration of zwitterionic molecules, which increases with decreasing spacing between positive and negative charges of the net-neutral moiety.^8^ In this context, ylides and *N*-oxides are interesting classes of zwitterions with the smallest possible distance (one bond length) between cation and anion.^9−13^*N*-Oxides are slightly basic molecules (p*K*_a_ of the corresponding protonated functional group 4 – 5) capable of forming strong hydrogen bonds.^14−17^ They are characterized by a large dipole moment and high polarity.^18^ Their properties make *N*-oxides appealing for various applications, including the use as oxidants, solvents, drugs and in material science.^19−22^ For these purposes, polymeric *N*-oxides have been grafted on several substrates.^11,21,23−35^ As noted above, charge density is a particularly important parameter of grafted polymeric *N*-oxides as it influences several surface properties, including their antifouling activity. Many grafted polymeric *N*-oxides are synthesized by grafting of tertiary amine-based monomers and their subsequent oxidation to the corresponding *N*-oxides by strong oxidants like H_2_O_2_ or peracids. These oxidations are slow and often not quantitative.^36,37^ Incomplete conversions can thus limit applications of the resulting materials. An easy method for quantification of *N*-oxide functionalities would thus be desirable to characterize the success of *N*-oxidation and evaluate the number of solvent accessible zwitterions. In addition to sophisticated analytical techniques like ToF-SIMS or XPS, the quantification of surface functional groups can be achieved with simple colorimetric assays as depicted in Scheme 1.^4,38,39^ These assays are easy to perform and do not need expensive and specialized instrumentation.^40^ In addition, dye adsorption does not measure the overall number of functional groups in the brush layer, but allows the quantification of solvent-exposed functional groups. The latter are relevant for most applications. In colorimetric assays, a dye is typically adsorbed to the surface via reversible charge interaction to the grafted polymer brushes (Scheme 1). After washing to remove excess unbound dye, desorption of the dye is achieved by ion exchange. The amount of desorbed dye, as quantified via spectroscopy, can be correlated to the surface charge.^41^ These assays have been shown to be useful for the quantification of grafted polymeric quaternary ammonium groups^4^ (Scheme 1A) and for grafted polysulfobetaines (Scheme 1B).^41^ The latter example is remarkable, because it includes the 1:1 interaction of a cationic dye (crystal violet, CV) and a zwitterionic sulfobetaine. Key interactions are most likely ion pairs between the sulfonic acid and the positively charged dye in combination with π-interactions. The zwitterionic structure of grafted *N*-oxides might also lead to electrostatic interaction with charged dyes, thus allowing the determination of the charge density of these molecules on surfaces.^42^

In this work, the interaction of charged dyes with grafted *N*-oxides has been investigated. The following questions are addressed: 1) Are established dye assays for other charged polymers transferable to poly-*N*-oxides? 2) How does the relatively high p*K*_a_-value of 4–5 influence the interaction of *N*-oxides with charged dyes? 3) Does the chemical reactivity of *N*-oxides affect the binding of dyes? The answers to these questions led to the development of a rapid assay for *N*-oxide quantification. On a more general level, our study reveals important details on the interaction of charged dyes with *N*-oxides and may thus be relevant for use of poly-*N*-oxides for wastewater purification.

## Experimental Section

### Chemicals and Materials

Polyethylene (PE) foils with a thickness of 750 μm were purchased from Goodfellow and were used as received. Vinylbenzyl chloride (90%), (vinylbenzyl)trimethylammonium chloride (VBTAC) (99%), dimethylamine solution (33% in ethanol), azo-bis(isobutylonitrile) (AIBN) (98%), hydrogen peroxide solution (30% v/v in water), (2-dimethylaminoethyl) methacrylate (99%) (MADMA), *N*-[3-dimethylamino)-propyl]-methacrylamide (99%) (MAADMA), cetyltrimethylammonium chloride (CTAC) (96%), acid fuchsin disodium salt (AF), crystal violet (CV), malachite green (MG) horseradish peroxidase, pyrogallol (99%) were purchased from Sigma-Aldrich. Trimethylamine *N*-oxide (TMAO) was purchased from BLD pharmatech. Fluorescein disodium salt was purchased from Alfa Aesar. All reagents were used without further purification. Phosphate-buffered saline (PBS) with a final concentration of 137 mm NaCl, 10 mm phosphate, 2.7 mm KCl and a pH adjusted to 8.0 was prepared as a stock solution. Crimp neck vials (N20, 10 mL volume) and crimp caps (N20, PTFE septum) for degassing were purchased from Macherey-Nagel GmbH (Düren, Germany).

### UV–Vis Spectroscopy

UV–vis spectra were obtained on a Genesys 10S spectrophotometer from Thermo Scientific (Waltham, USA) using Visionlite software for analysis.

### Infrared Spectroscopy

Infrared spectra were recorded with an attenuated total reflectance Fourier Transform infrared system (ATR-FTIR), model “IRAffinity-1S” from Shimadzu (Kyoto, Japan) using a “Quest” ATR accessory from Specac. The spectral range was set from 4000 cm^–1^ to 500 cm^–1^ with a resolution of 0.5 cm^–1^ in absorbance mode. The obtained spectra were processed with OriginPro 9 (2021) software.

### Contact Angle Measurements

Advancing contact angles were acquired with an OCA 20 goniometer from DataPhysics (Filderstadt, Germany) equipped with two automated dispensing units for different liquid probes, a high-speed video system with CCD-camera, measuring stage and halogen-lighting for static and dynamic contact angle measurements. For evaluation, independent triplicate measurements at three different points of the surface were done. Contact angles were measured with deionized H_2_O using the static sessile drop method with a dispensing volume of 5 μL. The dispensing rate of the automatic syringe was set at 1 μL·min^–1^. The obtained angle was calculated with the OCA software.

### NMR Spectroscopy

^1^H NMR measurements were carried out at 20 °C in 5 mm o.d. sample tubes with a Bruker Avance III HD 400 MHz (AV400, Bruker Biospin GmbH, Ettlingen, Germany). ^1^H NMR spectra were calibrated against deuterated methanol.

### Synthesis of Monomers

*N,N*-Dimethyl-1-(4-vinylbenzylamine) (VBDMA) and vinylbenzyl *N*-oxide (VBNOx) were prepared according to previously reported methods.^10,41^

### Synthesis of Poly(*N,N*-dimethyl-1-(4-vinylbenzylamine)) (pVBDMA)

*N,N*-Dimethyl-1-(4-vinylbenzylamine) (VBDMA) (3.00 g, 59 wt %) was added to a solution of azo-bis(isobutylonitrile) (AIBN) (75 mg, 1.5 wt %) in ethyl acetate (2.35 mL). The solution was degassed by Ar-purging for 30 min, then heated at 80 °C for 4 h. All volatile components were removed under reduced pressure. The resulting viscous polymer was dissolved in ethanol and purified by dialysis (MWCO = 12000 – 14000 Da) in a 1:1 mixture of ethanol/H_2_O. The purified polymer was obtained as an orange viscous liquid (0.84 g). ^1^H NMR (400 MHz, MeOD): δ [ppm] = 6.99 (2H, arH), 6.49 (2H, arH), 3.36 (2H, NCH_2_), 2.18 (6H, CH_3_) 1.74 (1H, CH), 1.46 (2H, CH_2_).

### Synthesis of Poly(vinylbenzyl *N*-oxide) (pVBNOx)

Azo-bis(isobutylonitrile) AIBN (20 mg) was added to VBDMA (1.00 g) and the mixture was purged with Ar for 15 min. The solution was then heated at 70 °C for 3 h. The crude product was stirred in aqueous H_2_O_2_ solution (30%, 6 mL) for 48 h. Residual H_2_O_2_ was decomposed by addition of activated charcoal as confirmed by H_2_O_2_ test strips (c(H_2_O_2_) < 0.5 mg/L). The colorless product was isolated as a white solid (0.67 g) after lyophilization. ^1^H NMR (400 MHz, MeOD): δ [ppm] = 7.24 (2H, arH), 6.51 (2H, arH), 4.29 (2H, NCH_2_), 3.04 (6H, CH_3_), 1.51 (3H, CH+CH_2_).

### Grafting from PE Foils

(Vinylbenzyl)trimethylammonium chloride grafted on PE (PE-pVBTAC),^43^*N,N*-dimethyl-1-(4-vinylbenzylamine) grafted on PE (PE-pVBDMA), *N,N*-dimethyl-1-(4-vinylbenzylamine-*N*-oxide) grafted on PE (PE-pVBNOx), 3-methacrylamido-*N,N*-dimethylpropan-1-amine oxide grafted on PE (PE-pMAANOx) and 2-(methacryloyloxy)-*N,N*-dimethylethan-1-amine oxide grafted on PE (PE-pMANOx) were prepared according to previously reported methods. The grafting yield of pVBNOx on PE was estimated from the layer thickness and the density of polystyrene to be 10.5 μg*cm^–2^ as reported before.^10^

### Horseradish-Peroxidase Assay

Horseradish-peroxidase assay was performed according to a previously reported method.^10^

### Determination of Extinction Coefficient of AF

Aqueous AF solution (1.00 mm) was diluted with an aqueous CTAC solution (0.1 wt %) to obtain four distinct solutions with concentration of 10.0 μm, 15.0 μm, 20.0 μm, and 30.0 μm, respectively. The absorbance of the solutions was measured at 556 nm. The molar extinction coefficient was calculated by the slope of the absorbance vs concentration plot. The same procedure was performed to determine the extinction coefficient of AF in H_2_O at pH 3 and pH 7. The pH of the solution was adjusted with aqueous HCl or NaOH solutions 1 m.

### Colorimetric Determination of Charge Density of Grafted *N*-oxides

Grafted foils with a total surface area of 1.0 cm^2^ were treated with 2.0 mL of AF solution (1.0 wt %). The initial pH of the dye solution was adjusted with aqueous HCl or NaOH solutions 1 m to the desired value using a Mettler Toledo pH meter. The contents were shaken at 100 rpm using an orbital shaker (VWR) for a fixed amount of time at 20 °C. The foils were then removed and briefly rinsed with deionized H_2_O. The foils were immersed in deionized H_2_O or aqueous HCl solution at pH 3 for 10 min with ultrasonication to remove residual dye. For desorption of the adsorbed dye, the foils were treated with 5.0 mL of an aqueous CTAC solution (0.1 or 1 wt %). The pH of the desorption solution was adjusted with aqueous 1 m HCl to the desired value using a Mettler Toledo pH meter. After shaking at 100 rpm for 1 h at 20 °C, the pH of the desorption solution was adjusted to pH 3 with aqueous 1 m HCl solution. The absorbance of the resulting solution was measured at 556 nm. The absorption of a solution obtained by the same procedure from a nonmodified PE foil was subtracted as a blank value. The resulting UV–vis absorbance was correlated to the concentration of desorbed AF using the Beer–Lambert law:
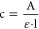


A = measured absorbance

ε = extinction coefficient of AF in CTAC (38 mm^–1^·cm^–1^)

l = optical path length [cm]

c = concentration of desorbed AF [mol/L].

All measurements were performed in triplicate.

### Fluorescein Assay

The charge density of PE-pVBTAC foils was determined according to a previously reported method employing fluorescein.^43^

### Electrokinetic ζ-Potential Measurements

The surface zeta potential (ζ-potential) was determined as streaming potential using an electrokinetic analyzer Surpass (Anton Paar, Graz, Austria). The measurements were carried out using an adjustable gap cell in which two samples with a rectangular size of 1 cm × 2 cm were clamped vis-á-vis with a micro slit of 110 μm in between. For each measurement, the starting conductivity of each measurement was set to 17 μS/m with KCl as electrolyte. The pH was adjusted from 9.5 to 2.5 stepwise with automatic pH titration by adding 0.05 M HCl. Presented values of ζ potentials were determined as mean value of four measurements for each pH step.

### Reactivity between TMAO and AF

Ten μL of aqueous AF solution (3.4 mm, pH 5) were added to 990 μL of aqueous TMAO solution (69 μm and 515 μm, pH 8). A control solution was prepared by adding 10 μL of aqueous AF solution (3.4 mm, pH 5) to 990 μL of aqueous NaOH solution (pH 8). The absorbance of the resulting solutions was measured at 546 nm for 30 min at intervals of 30 s.

### Reactivity between VBNOx and Triarylmethane Dyes

Ten μL of aqueous dye solution (17 mm) were added to 5.0 mL of aqueous VBNOx solution (34 mm) (VBNOx-dye). An aqueous solution of the dye (Ctrl) was used as a positive control. The UV–vis spectra of the resulting solutions were recorded in the range of 300–650 nm after 60 min of the dye adding. Triarylmethane dyes used in this study included AF, CV, and MG. The percentage of absorbance reduction was calculated from the absorbance values at the wavelength of highest absorption for each dye (546 nm for AF, 590 nm for CV, and 617 nm for MG), using the following formula:
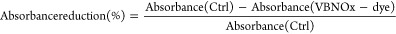


## Results and Discussion

### Synthesis of Charged Polymer Brushes

**Scheme 2:**
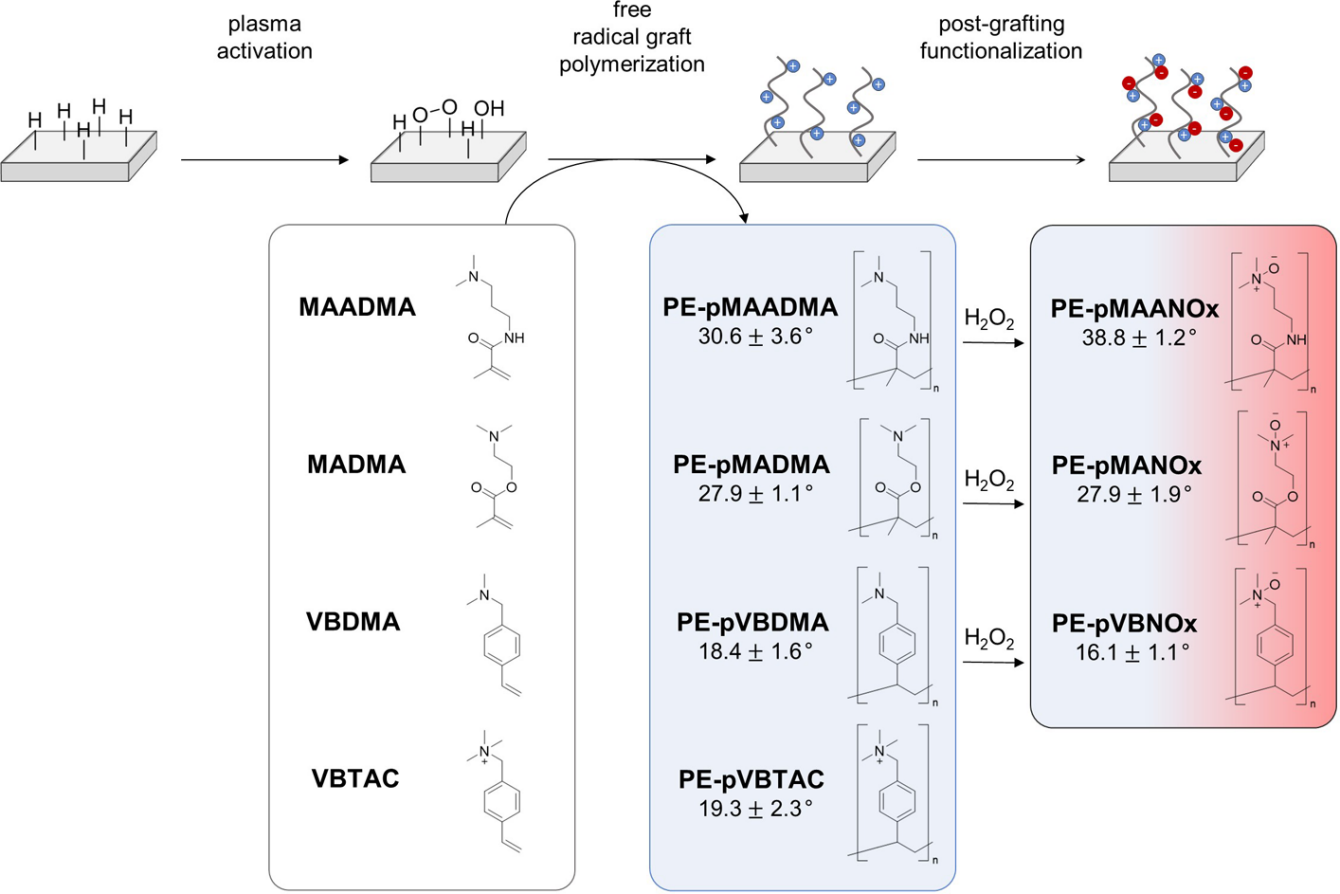
Synthetic Scheme, Structures and Water Contact Angles of Grafted Charged Polymers Advancing contact angles [°] are given as mean values of three measurements ± standard deviation (SD).

This study was based on *N*-oxide polymer brushes grafted to polyethylene (PE). PE was chosen because it is a widely used thermoplastic polymer prepared by cost-effective manufacturing processes^44^ and has a wide range of applications in the food and healthcare sector.^45,46^ In this context, numerous studies have explored the surface modification of PE to develop antibacterial and antifouling properties.^47,48^ Among other approaches, the grafting of *N*-oxide polymers to PE has also been described.^10^ In this report, *N*-oxide polymer brushes were grafted to PE by an established three-step protocol depicted in Scheme 2.^10^ Briefly, PE foils were activated by atmospheric air plasma treatment followed by free radical polymerization with the appropriate monomer and AIBN. PE-pVBDMA, PE-pMADMA, PE-pMAADMA and PE-pVBTAC were obtained by this procedure. The brush polymers of PE-pVBDMA, PE-pMADMA and PE-pMAADMA bear tertiary amine groups which were subsequently oxidized to the corresponding *N*-oxides with H_2_O_2_ to give PE-pVBNOx, PE-pMANOx and PE-pMAANOx. All materials obtained by this procedure were extensively washed with water under ultrasonication for removal of any noncovalently bound material. In addition, PE-pVBNOx, PE-pMANOx and PE-pMAANOx were treated with aqueous 1 m NaOH solution for 48 h to remove residual H_2_O_2_. The complete removal of H_2_O_2_ was confirmed via a horseradish peroxidase assay.^10^ Full characterization of the resulting materials by XPS, ToF-SIMS, IR, and goniometry has been reported before and confirmed the presence of the anticipated polymers in dense brush layers of ∼ 50–100 nm thickness.^10^

### Colorimetric Analysis of the Charge Density

The solvent accessible surface charge density of the materials is an important parameter for many applications including contact-killing of bacteria, wastewater purification (adsorption of ions) or antifouling. However, this parameter is hard to analyze with standard methods for surface analysis. A colorimetric assay was therefore developed, according to the general method depicted in Scheme 1C.

**Figure 1:**
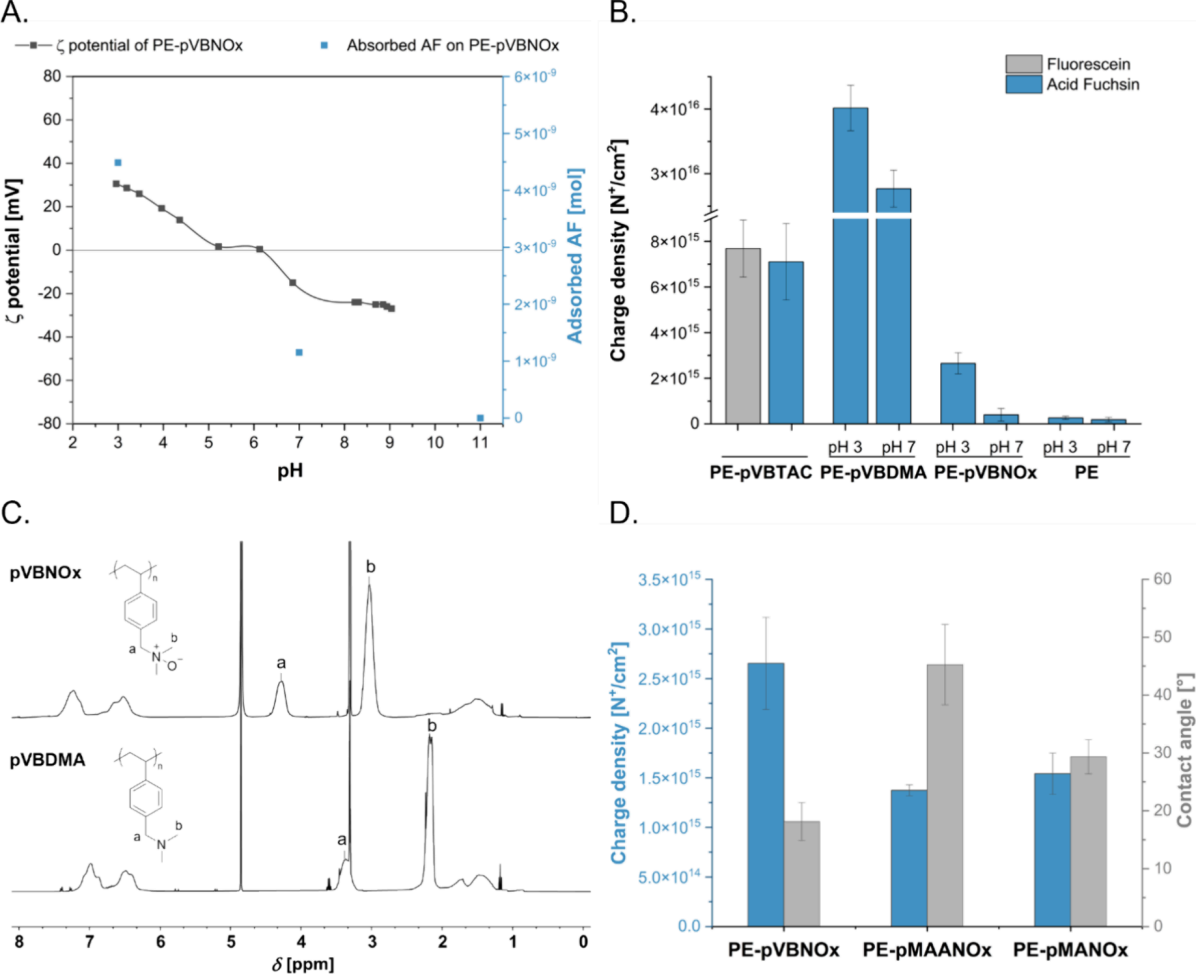
A) Surface zeta potential and amount of adsorbed AF on PE-pVBNOx at different pH values. B) Comparison of the charge density of PE-pVBTAC as measured with fluorescein or AF, and charge density of PE-pVBDMA, PE-pVBNOx, and PE as measured with AF at pH 3 and 7. C) Partial ^1^H NMR spectra (400 MHz, MeOD) of pVBNOx (top) and pVBDMA (bottom). D) Correlation between charge density and contact angle for PE-pVBNOx, PE-pMAANOx and PE-pMANOx. Conditions: 1 cm^2^ PE test specimens were immersed for 1 h in 2 mL of selected staining solution: aqueous AF solution (1 wt %, pH 3 adjusted with HCl 1 m or pH 7 adjusted with NaOH 1 m) or aqueous fluorescein solution (1 wt %, pH 8). Specimens were washed with an aqueous solution with ultrasonication for 10 min, followed by dye desorption with 5 mL CTAC solution (0.1 wt % pH 7). For AF, the pH of the desorption solution was then adjusted to 3 with HCl 1 m and the absorbance was measured at 556 nm. For fluorescein, the absorbance was measured at 501 nm. The molar equivalents of the dye and the charge density were calculated assuming a 1:1 binding of dye to *N*-oxide or ammonium group.

The surface charge of grafted *N*-oxides depends on the pH of the medium used for immersion. As mentioned above, alkyl-substituted *N*-oxides have p*K*_a_ values of 4–5, leading to protonation (N^+^–OH) and thus a positive surface charge at low pH. At high pH, deprotonation results in a net-neutral charge of zwitterionic poly-*N*-oxides (N^+^-O^–^). These properties are reflected by the pH-dependent surface zeta potential of polymeric *N*-oxides on bulk material, which is exemplarily depicted for PE-pVBNOx in Figure 1A. The surface zeta potential of PE-pVBNOx is positive at acidic pH due to the protonation of surface *N*-oxides and the formation of the corresponding hydroxylammonium groups. It is neutral between pH 5–6 indicating the presence of the zwitterionic *N*-oxide groups on the surface, and negative at more alkaline pH due to the increased accumulation of hydroxide ions on the surface.^10,49^ This pH-dependent charge of grafted *N*-oxides allows the reversible adsorption of charged dyes (and any other charged molecule) through electrostatic interactions. pH values below ∼ 4 (p*K*_a_ of surface *N*-oxides) lead to a positive surface charge, facilitating electrostatic interaction with anionic dyes. At higher pH values (pH > p*K*_a_), deprotonation of *N*-oxides occurs, resulting in an increased number of zwitterionic sites. The presence of these negative charges on the surface repels anionic dyes preventing their adsorption to the surface. In addition, the excess of hydroxide in alkaline solution competes with the anionic dye molecules for adsorption to the surface.^50^

Colorimetric determination of charged functional groups relies on the noncovalent adsorption of dyes to the surface through charge interaction between the dye and the surface-bound *N*-oxide groups. In theory, zwitterionic *N*-oxides allow charge interactions with both, cationic and anionic dyes. A simple colorimetric analysis is particularly interesting for polymeric *N*-oxides: typically, grafted *N*-oxides are prepared by graft polymerization of suitable acrylate or styrene derivatives bearing tertiary amine groups and a postgrafting oxidation to the corresponding *N*-oxide brush polymers. This oxidation step may not yield complete conversion. Therefore, employing a cationic dye to selectively address the negatively charged oxygen atoms appears to be the most effective strategy to target *N*-oxides. Crystal violet (CV), for example, is a cationic dye previously used for the surface charge determination of other zwitterionic polymers such as polysulfobetaines.^41^ However, staining of grafted polymeric *N*-oxides (at pH 7) with CV was unsuccessful. This finding might be due to the minimal spacing of charges (just one bond length) in *N*-oxides. The polysulfobetaines tested before had a significantly longer charge spacing (3 carbon spacer). This lack of spacing leads most likely to electrostatic repulsion of the cationic dye and the positively charged nitrogen in *N*-oxides like PE-pVBNOx. The known colorimetric analysis of sulfobetaines can therefore not be transferred to grafted *N*-oxides.

Compared to sulfobetaines (p*K*_a_ of the sulfonic acid ∼ 1), *N*-oxides have a higher p*K*_a_ of 4–5 allowing protonation at pH values lower than 4–5.^36^ Anionic (acidic) dyes can thus be used for the quantification of grafted *N*-oxides through electrostatic interactions with the protonated and thus positively charged surface at moderately acidic pH values. It is mandatory for the p*K*_a_ of acidic groups of the dye to be significantly lower than 4 to match the p*K*_a_ of the *N*-oxides and open a pH range with a positive charge of the surface *N*-oxides and a negative charge of the dye. Fluorescein, for example, is an acidic dye commonly employed to determine the charge density of ammonium groups on surfaces.^4^ However, this dye by bearing a carboxylic acid with a p*K*_a_ of ∼ 5 lacks a negative charge at pH values <4 which is required for the protonation of *N*-oxides.^51^ Sulfonic acids, in contrast, are stronger acids with much lower p*K*_a_ values than carboxylic acids. Acid fuchsin (AF), a sulfonylated triarylmethane dye, was therefore selected as an anionic dye. It is known to have ideal acid/base properties for the anticipated charge interaction with *N*-oxides because it carries a single negative charge at slightly acidic pH values.^52^

The adsorption of AF to PE-pVBNOx was tested at pH values of 3, 7, and 11 as depicted in Figure 1A. The pristine PE foils used for comparison showed no dye adsorption at pH 3–11. A pH-dependent adsorption of AF to the polymeric *N*-oxides was observed in these experiments. Treatment of PE-pVBNOx with an aqueous solution of AF at pH 3 led to adsorption of the dye, accompanied by red staining of the test foils (see Figure S1, A). In contrast, the PE-pVBNOx specimens remained colorless after treatment with AF solution at pH 7 (see Figure S1, B). These experiments confirmed qualitatively the pH-dependent adsorption of AF to surface grafted *N*-oxides. However, for quantification, the adsorbed dye must be completely desorbed from the surface and analyzed photometrically. Each test specimen was therefore washed extensively after adsorption of AF. This washing step ensures the complete removal of excess dye from the surface while preserving the integrity of the N^+^-dye^–^ ion pairs formed upon staining. Washing was performed by treatment of the test specimens with aqueous HCl (pH 3) with ultrasonication for 10 min.

Subsequent desorption of the dye was achieved with an aqueous cetylammonium chloride (CTAC) solution at pH 7. The successful desorption of AF was clearly visible and led to the formation of colorless PE-pVBNOx test specimens. Complete desorption of AF can be attributed to two factors: the presence of CTAC, which acts as a counterion for the anionic dye, and the pH of the desorption solution. Since the pH exceeds the p*K*_a_ value of the *N*-oxides, the surface becomes more negatively charged which enhances the repulsion of the anionic dye. The resulting desorption solution contains the released dye in its benzenoid colorless form.^53^ Colorimetric quantification of AF was therefore performed after addition of 1 m HCl to the desorption solution at pH 3. At low pH, the quinoid form of AF is restored and AF has a high molar absorption coefficient (ε) of 38925 m^–1^ cm^–1^, resulting in a strong absorption at 556 nm (λ_max_).

Assuming a 1:1 binding of AF with positively charged hydroxylammonium groups, a solvent accessible charge density of 2.65 × 10^15^ N^+^/cm^2^ was calculated for PE-pVBNOx from the colorimetric analysis of the desorption solution. It is notable that repeated adsorption/desorption of AF with the same PE-pVBNOx test specimen revealed almost identical charge density values (see Figure S2) demonstrating the reversibility of the adsorption/desorption process.

The 1:1 binding of AF to *N*-oxides was verified by comparison to the dye adsorption of purely cationic polyammonium brushes of PE-pVBTAC for two reasons: First, The poly(quaternary ammonium) brushes of PE-pVBTAC resemble the positively charged nitrogen of protonated *N*-oxides. Second, quaternary ammonium groups have been demonstrated to form 1:1 ion pairs with fluorescein as an anionic dye in previous studies.^54^ The charge density of PE-pVBTAC derived from the colorimetric analysis with fluorescein was therefore compared to the value obtained with AF on the same material and was found to be almost identical (Figure 1B). In addition, the number of solvent accessible charges derived from AF-binding to PE-pVBNOx (2.65 × 10^15^ N^+^/cm^2^) compares well with the charge density of PE-pVBTAC (7.11 × 10^15^ N^+^/cm^2^) prepared by the same general synthetic scheme. It is notable that the charge density of PE-pVBDMA, the synthetic precursor of PE-pVBNOx, was significantly higher. The value of 4.02 × 10^16^ N^+^/cm^2^ for PE-pVBDMA is approximately an order of magnitude higher than that of PE-pVBNOx (2.65 × 10^15^ N^+^/cm^2^). This reduced charge density for PE-pVBNOx is most likely a result of the oxidation step which might reduce the number of solvent accessible sites in the polymer chains due to the additional OH groups. An alternative explanation, the loss of eventually noncovalently bound polymer from the surface is unlikely. The materials have been washed excessively with solvent and the graft polymers have been shown to be highly stable against polymer washout before.^10^

PE-pVBDMA was oxidized with H_2_O_2_ to PE-pVBNOx. The conversion of tertiary amine groups to the corresponding *N*-oxides is known to be quite slow and incomplete conversions might therefore be expected.^18^ The efficiency of the oxidation can be estimated by analyzing the adsorption of AF to PE-pVBNOx at different pH values: At pH 3 both, *N*-oxide and remaining tertiary amine groups (left through incomplete oxidation of PE-pVBDMA), are positively charged and thus bind AF. However, at pH 7, only the remaining tertiary amine groups bind AF, as the zwitterionic *N*-oxides do not interact with the dye. A comparison of the charge density values obtained at these two pH values (pH 3: 2.65 × 10^15^ N^+^/cm^2^, pH 7: 3.95 × 10^14^ N^+^/cm^2^) reveals approximately 90% oxidation efficiency. This aligns with the NMR-analysis of the same oxidation protocol applied to water-soluble nongrafted pVBDMA, which revealed almost complete conversion to pVBNOx (Figure 1C). Both compounds can be easily distinguished by the downfield shift of signals for the benzyl methylene and the methyl protons in pVBNOx compared to pVBDMA.

The impact of staining time on the outcome of AF adsorption was investigated from 1–18 h. The amount of AF adsorbed during the staining step slightly increases with longer contact times, from 3.41 × 10^–9^ mol/cm^2^ (1 h) to 4.21 × 10^–9^ mol/cm^2^ (18 h, see Figure S3). This can be attributed to the swelling of the polymer brushes, which leads to an increased number of available sites for dye adsorption.^55^ However, the effect is quite small and has only a minor influence on the calculated charge density. A 1 h staining time of the materials in AF solution is therefore recommended to determine the surface accessible charges, for practical reasons.

The colorimetric analysis of AF adsorption allows the determination of the charge density on surfaces, making it a valuable method to assess the success of brush polymer synthesis and to estimate surface properties for example for antifouling applications. The assay was therefore used to evaluate the charge density of three different grafted polymeric *N*-oxides on PE (Figure 1D). All three *N*-oxides were synthesized by the same protocol via polymerization of common monomers. The resulting charge densities were 2.65 × 10^15^ N^+^/cm^2^ for PE-pVBNOx, 1.37 × 10^15^ N^+^/cm^2^ for PE-pMAANOx and 1.64 × 10^15^ N^+^/cm^2^ for PE-pMANOx. The charge densities correlate well with the corresponding water contact angles of these materials (Scheme 2), because higher charge densities correspond to lower water contact angles. These results align also well with the antifouling effect of the materials. In a previous comparative study,^10^ PE-pMAANOx showed a weaker antifouling effect than PE-pMANOx and PE-pVBNOx reflecting the higher charge density of the latter two materials. It should be noted that the observed difference in antifouling efficiency does not reflect the intrinsic antifouling properties of the *N*-oxides. The effect is rather due to the different grafting yields achieved for the three monomers and the protocol used. The adsorption of AF via electrostatic interactions is thus useful in the assessment of charge density of grafted *N*-oxides, which can be correlated to their antifouling activity. This method is also a valuable tool to verify the success of the grafting process and might also be used to improve its efficiency. The effect of reaction parameters, such as reaction time and monomer concentration, on the grafting efficiency can easily be monitored by this technique, as demonstrated for selected examples in Figure S4.

The optimal assay conditions are the following: test specimens are treated with aqueous AF solution (1.0 wt %) at pH 3 (adjusted with HCl) for 1 h at 20 °C. The test specimens are then removed and washed with aqueous HCl solution (pH 3) with ultrasonication. For desorption of the dye, the test specimens are treated with a desorption solution containing CTAC (0.1 wt %, pH 7) for 1 h at 20 °C. After removal of the test specimens, the pH of the desorption solution is adjusted to pH 3 with aqueous 1 m HCl solution and the absorbance of the resulting solution is measured at 556 nm.

### Covalent Binding of N-oxides to Triaryl Methane Dyes

Polymeric *N*-oxides are often considered to be of low chemical reactivity. This property is important for applications requiring biocompatibility and durability. Grafted polymeric *N*-oxides, for example, have stealth properties in biological systems and are used for antifouling applications.^19^ However, the reactivity of *N*-oxide functionalities depends strongly on their chemical structure. In fact several derivatives, which are known to be quite reactive, are used as oxidation reagents in synthesis.^56,57^*N*-Oxides that are also known to undergo various other reactions such as eliminations or rearrangements are thus not unreactive *per se*.^58^ The binding processes between surface *N*-oxides and dyes might not only be driven by charge interactions but also by others, such as hydrogen bonding, π-stacking or covalent binding depending on the properties of the dye.^42,59^ The latter point is noteworthy because *N*-oxides can act as *O*-nucleophiles toward various electrophiles.^18^ They may thus be able to react with electron-deficient triarylmethane dyes like AF. The potential impact of covalent binding between dyes and grafted *N*-oxides on the outcome of AF adsorption was therefore examined.

**Figure 2:**
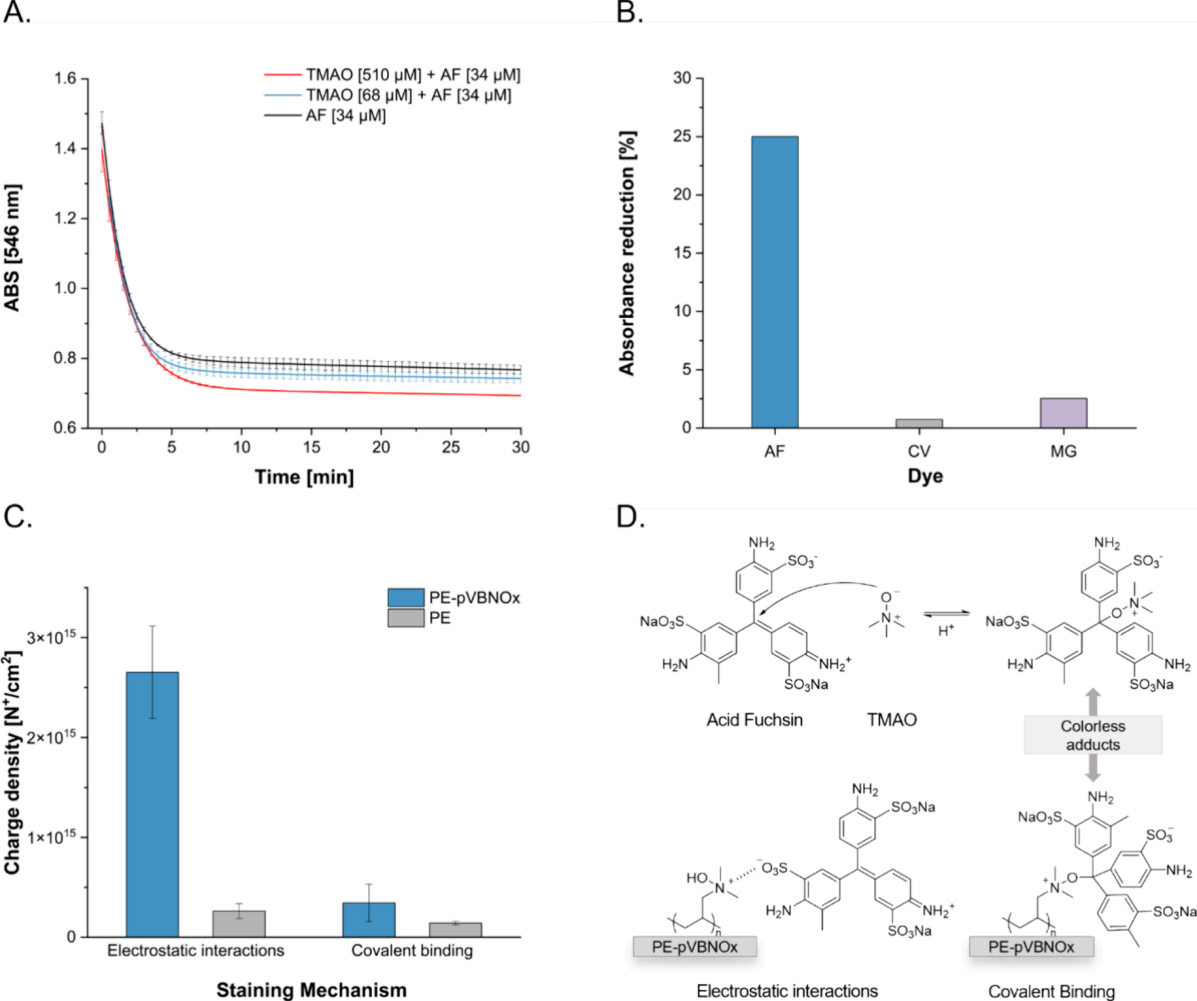
A) Reactivity of AF with TMAO at different concentrations at pH 8 as measured by absorbance at 546 nm. B) Reduction of absorbance for reaction of VBNOx with triarylmethane dyes. C) Charge density values of PE-VBNOx and PE based on the different staining mechanism. For electrostatic interactions: foils were treated in 2 mL of aqueous AF solution (1 wt %, pH adjusted to 3 with aqueous 1 m HCl) for 1 h. Specimens were washed with aqueous HCl (pH 3) with ultrasonication for 10 min, followed by dye desorption with 5 mL CTAC solution (0.1 wt % pH 7). The pH of the desorption solution was then adjusted to 3 with HCl 1 m and the absorbance was measured at 556 nm. For covalent interactions: foils were treated in 2 mL of aqueous AF solution (1 wt %, pH adjusted to 7 with aqueous 1 m NaOH solution) for 1 h. Specimens were washed with deionized H_2_O (pH 7) with ultrasonication for 10 min, followed by dye desorption with 5 mL CTAC solution (0.1 wt %, pH adjusted to 3 with aqueous 1 m HCl). The pH of the desorption solution was then adjusted to 3 with 1 m HCl and the absorbance was measured at 556 nm. Results are given as mean values of three measurements ± standard deviation. D) Electrostatic and covalent interaction of *N*-oxides and AF.

Triarylmethane dyes react with nucleophiles in aqueous neutral and alkaline solutions to form colorless covalent adducts. This reaction is reversible at low pH values and the color of the dye can thus be retained in acidic solution.^53,60^ The reactivity of *N*-oxides and triarylmethane dyes was investigated with trimethylamine *N*-oxide (TMAO), a commercially available *N*-oxide, and AF, an electron-deficient triarylmethane dye. Aqueous TMAO solutions of different concentrations were treated with aqueous AF solution at pH 8, and the reaction between AF and *N*-oxide was monitored by measuring the absorbance at 546 nm over time (Figure 2A). Upon reaction with the nucleophile (TMAO), the conjugate system of the electrophilic dye is disrupted, resulting in a colorless product (Figure 2D). Consequently, the absorbance at 546 nm decreases. For comparison, a control solution was prepared by diluting an aqueous AF solution (3.4 mm, pH 5) with an aqueous NaOH solution, in order to match the pH value of the aqueous TMAO solutions (i.e., pH 8). Under these conditions, OH^–^ ions act as nucleophiles, reacting with the dye and causing a measurable decrease in absorbance. However, when AF was added to the aqueous TMAO solutions, the decrease in absorbance was significantly higher than that observed in the control solution and was proportional to the TMAO concentration. These findings suggest the formation of covalent bonds between TMAO and the triarylmethane dye.

VBNOx has a similar reactivity (compared to TMAO) with AF, resulting also in a decrease in absorbance of the resulting solution after 60 min (Figure 2B). Notably, reactivity of *N*-oxides was observed with electron-deficient triarylmethane dyes like AF only. More electron-rich dyes, such as Crystal violet (CV) and Malachite green oxalate (MG) (Figure 2B, for full UV–vis spectra see Figure S5) did not form covalent adducts with *N*-oxides. This reactivity trend parallels thus the electron-deficiency of the dyes tested. It is notable that a similar trend was observed for bleaching of different triarylmethane dyes in the presence of amino acids and proteins.^60^

For the charge density measurement, AF is desorbed from grafted *N*-oxides by treatment with an aqueous CTAC solution at pH 7. These conditions may favor the covalent binding of AF to the *N*-oxides, thus compromising the outcome of the analysis. As a result, covalently bound AF molecules may remain on the surface rather than being desorbed, potentially leading to an underestimation of the calculated charge density. In order to determine if covalent binding at neutral pH significantly influences the desorption step, experiments were conducted using aqueous CTAC solutions (1 wt %) at pH 3 and at pH 7. A 10-fold higher amount of CTAC was used in these experiments to ensure efficient dye desorption through ion exchange even at low pH.

Slightly higher charge density values were obtained when desorption was performed at pH 7 compared to pH 3 (see Figure S6). These findings suggest that covalent binding of AF to the surface *N*-oxides does not significantly impact the desorption process at pH 7, confirming the positive impact of neutral pH on the electrostatic repulsion of the zwitterionic *N*-oxides on the surface and the anionic dye.

Moreover, the development of an assay based on the covalent addition of AF to grafted *N*-oxides, rather than relying on electrostatic interactions, was also explored. Theoretically, the two approaches should yield comparable results: at pH 3, staining occurs via electrostatic interactions between the anionic dye and the protonated *N*-oxides, whereas at pH 7, covalent binding should take place between the electrophilic dye and the nucleophilic oxygen of the *N*-oxides (Figure 2 D). The key advantage of the latter approach is its reliance on the nucleophilicity of *N*-oxides, a unique property that would allow the distinction from their parent tertiary amine groups. Therefore, the charge density was determined by staining PE-pVBNOx foils with an aqueous AF staining solution (1 wt %) at pH 7, creating favorable conditions for the covalent binding of AF to the foils. This was followed by washing in deionized water (pH 7) and desorption in an aqueous CTAC solution (1 wt %) at pH 3, where covalent binding on the surface cannot occur. In this context, a higher concentration of the CTAC solution was necessary to prevent the electrostatic adsorption of the anionic AF to the protonated *N*-oxide groups on the surface. The obtained charge density was 3.44 × 10^14^ N^+^/cm^2^ and thus significantly lower than the charge density of PE-pVBNOx measured using the same procedure but employing AF at pH 3 as staining solution (2.06 × 10^15^ N^+^/cm^2^) (see Figure S7). These results suggest that covalent interactions between *N*-oxides and AF cannot be used for the determination of charge density under these conditions (Figure 2C). Moreover, the effect of covalent binding is negligible under the conditions tested and does not compromise the charge density measurement with AF.

## Conclusions

Polymeric *N*-oxides have recently attracted interest as materials for antifouling, drug delivery, wastewater purification and electronic devices. The number of solvent accessible N^+^-O^–^ functionalities is an important parameter of polymeric *N*-oxides with relevance for many fields of application. In this study, the interaction of charged dyes with grafted *N*-oxides was studied and a new method for the quantification of solvent accessible N^+^-O^–^ functionalities developed.

Grafted polymeric *N*-oxides show a different capacity for adsorption of charged dyes compared to other polyzwitterionic materials. Established colorimetric assays for polysulfobetaines rely on adsorption of the basic dye CV, which does not stain *N*-oxides. Instead, the proposed colorimetric adsorption/desorption assay for *N*-oxides relies on the reversible electrostatic interaction with AF, an acidic dye. Adsorption and desorption of AF are facilitated through the pH-responsive properties of grafted *N*-oxides (p*K*_a_ ∼ 4–5) and the strongly acidic dye AF (p*K*_a_ ∼ 1). It is a rapid and convenient method for the estimation of grafted *N*-oxide functionalities with common laboratory equipment. Application to various grafted *N*-oxides, revealed charge densities from 1–3 × 10^15^ N^+^/cm^2^.

Surface grafted polymers and dissolved *N*-oxides of low molecular weight bind covalently to electrophilic dyes, such as AF and are therefore not unreactive *per se*. In fact, the decrease in adsorption of AF upon treatment with TMAO was attributed to the disruption of the conjugated system of the dye resulting from the covalent binding of the *N*-oxide to the dye. Similar reactivity was observed with the styrene-based *N*-oxide VBNOx and AF. Covalent bond formation relies on nucleophilic displacement and is restricted to the reaction of *N*-oxides with electron-deficient triarylmethane dyes like AF. More electron rich dyes like CV and MG do not form covalent adducts with *N*-oxides. However, even with AF the reaction is quite slow and does not compromise the measurement of charge density as mentioned above. Covalent binding of AF to *N*-oxides at neutral pH, while detectable in solution, has only a negligible impact on dye desorption from surfaces.

In summary, this new assay for *N*-oxide functionality is a powerful tool to assess the efficiency of grafting processes and postgrafting oxidations of tertiary amine groups to *N*-oxides. It allows the differentiation between *N*-oxides and their tertiary amine precursors, making use of their pH-responsive properties. In a more general context, the adsorption/desorption capacity of polymeric *N*-oxides for charged dyes depends strongly on the pH and can involve electrostatic and covalent interactions. These findings are also relevant for nanofiltration of dyes from wastewater and can explain the efficiency of high-surface area materials (e.g., membranes) bearing grafted polymeric *N*-oxides in this context.^16,42,61,62^

## Supporting Information Available

Qualitative effect of different pH values of the staining solution time on the quantity of adsorbed AF on PE-pVBNOx and PE; charge density of PE-pVBNOx, PE-pMAANOx, and PE-pMANOx after the first and second cycle of the assay; effect of staining time on the quantity of adsorbed AF on PE-pVBNOx; effect of grafting time on charge density of PE-pVBNOx; fading of the color of aqueous triarylmethane dye solutions by VBNOx; effect of desorption solution pH on PE-pVBNOx; effect of staining solution pH on charge density of PE-pVBNOx (PDF)

## Abbreviations

AF: Acid fuchsin
AIBN: Azo-bis(isobutylonitrile)
CTAC: Cetyltrimethylammonium chloride
CV: Crystal violet
HRP: Horseradish peroxidase
MAADMA: *N*-[3-dimethylamino)-propyl]-methacrylamide
MADMA: (2-Dimethylaminoethyl) methacrylate
MG: Malachite green
PBS: Phosphate-buffered saline
PE-pMAANOx: 3-Methacrylamido-*N*,*N*-dimethylpropan-1-amine oxide grafted on PE
PE-pMANOx: 2-(Methacryloyloxy)-*N*,*N*-dimethylethan-1-amine oxide grafted on PE
PE-pVBDMA: *N*,*N*-dimethyl-1-(4-vinylbenzylamine) grafted on PE
PE-pVBNOx: *N*,*N*-dimethyl-1-(4-vinylbenzylamine-*N*-oxide) grafted on PE
PE-pVBTAC: (Vinylbenzyl)trimethylammonium chloride grafted on PE
pVBDMA: Poly(*N,N*-dimethyl-1-(4-vinylbenzylamine))
pVBNOx: Poly(vinylbenzyl *N*-oxide)
SDS: Sodium dodecyl sulfate
TMAO: Trimethyl amine *N*-oxide
VBDMA: *N*,*N*-dimethyl-1-(4-vinylbenzylamine)
VBTAC: (Vinylbenzyl)trimethylammonium chloride.
