# Supplementary material for: Interaction of Grafted Polymeric N-oxides with Charged Dyes
Source: Langmuir. 2025 Apr 25;41(17):11136–46. doi: 10.1021/acs.langmuir.5c00923 (PMC12060641; doi:10.1021/acs.langmuir.5c00923)
Supplement: Supplementary file 1 — la5c00923_si_001.pdf [file la5c00923_si_001.pdf]

Supporting Information

Interaction of grafted polymeric *N*-oxides with charged dyes

*Erica Moretto, Michelle Kobus and Wolfgang Maison**

Universität Hamburg, Department of Chemistry, Bundesstrasse 45, 20146 Hamburg, Germany

* Corresponding author: Wolfgang Maison. Email: wolfgang.maison@uni-hamburg.de

Table of content

Figure S1. Qualitative effect of different pH values of the staining solution time on the quantity of adsorbed AF on PE-pVBNOx and PE

Figure S2. Charge density of PE-pVBNOx, PE-pMAANOx and PE-pMANOx after the first and second cycle of the assay

Figure S3. Effect of staining time on the quantity of adsorbed AF on PE-pVBNOx

Figure S4. Effect of grafting time on charge density of PE-pVBNOx.

Figure S5. Fading of the color of aqueous triarylmethane dye solutions by VBNOx.

Figure S6. Effect of desorption solution pH on PE-pVBNOx

Figure S7. Effect of staining solution pH on charge density of PE-pVBNOx.

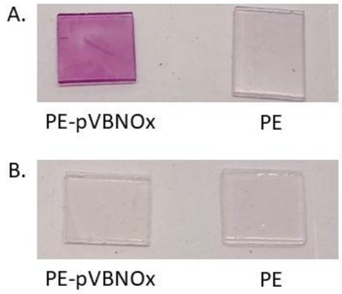

Figure S1. Qualitative effect of different pH values of the staining solution on the quantity of adsorbed AF on PE-pVBNOx and PE. A. Foils were treated in 2 mL of aqueous AF staining solution (1.0 wt%, pH adjusted to 3) for 1 h. The foils were then immersed in an aqueous washing solution at pH 3 for 10 min with ultrasonication. B. Foils were treated in 2 mL of aqueous AF staining solution (1.0 wt%, pH adjusted to 7) for 1 h. The foils were then immersed in an aqueous washing solution at pH 3 for 10 min with ultrasonication.

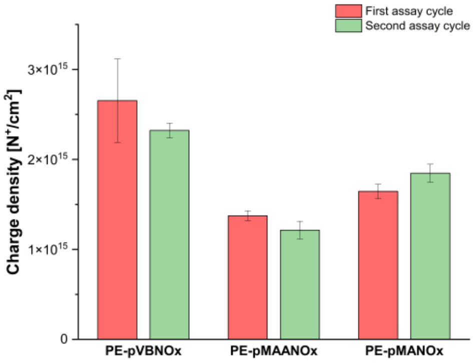

Figure S2. Charge density of PE-pVBNOx, PE-pMAANOx and PE-pMANOx after the first (red) and second (green) cycle of the assay. Foils were treated in 2 mL of aqueous AF solution (1 wt%, pH adjusted to 3) for 1 h. The foils were then immersed in an aqueous washing solution at pH 3 for 10 min with ultrasonication. For desorption of the dye, the foils were treated in 5 mL of aqueous CTAC solution (0.1 wt%) for 1 h at 20 °C. Subsequently, the pH of the desorption solution was

adjusted to pH 3 with aqueous HCl solution 1 M. The same process was repeated on the same foils for a second time. Results are given as mean values ± standard deviation (SD) $n = 3$.

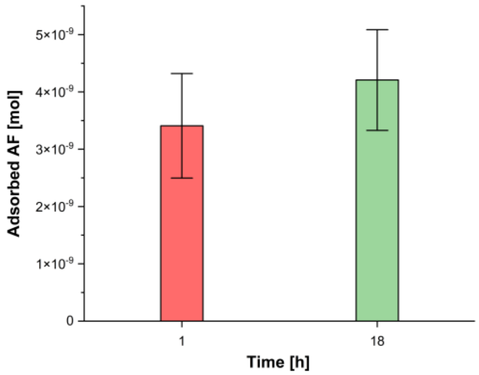

Figure S3. Effect of staining time on the quantity of adsorbed AF on PE-pVBNOx. Foils were treated in 2 mL of aqueous AF staining solution (1.0 wt%, pH adjusted to 3) for 1 h or 18 h. The foils were then immersed in an aqueous washing solution at pH 3 for 10 min with ultrasonication. For desorption of the dye, the foils were treated in 5 mL of aqueous CTAC solution (0.1 wt%) for 1 h at 20 °C. Subsequently, the pH of the desorption solution was adjusted to pH 3 with aqueous HCl solution 1 M. Results are given as mean values ± standard deviation (SD) $n = 3$.

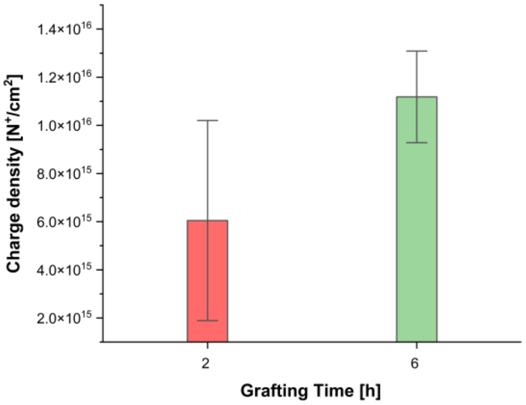

Figure S4. Effect of grafting time on charge density of PE-pVBNOx. The foils were prepared following a previously reported method, the polymerization was performed at 85 °C for 2 h or 6 h. Charge density values were obtained by treating the foils in 2 mL of aqueous AF solution (1 wt%, pH adjusted to 3) for 1 h. The foils were then immersed in an aqueous washing solution at pH 3 for 10 min with ultrasonication. For desorption of the dye, the foils were treated in 5 mL of aqueous CTAC solution (0.1 wt%) for 1 h at 20 °C. Subsequently, the pH of the desorption solution was adjusted to pH 3 with aqueous HCl solution 1 M. Results are given as mean values ± standard deviation (SD) $n = 3$.

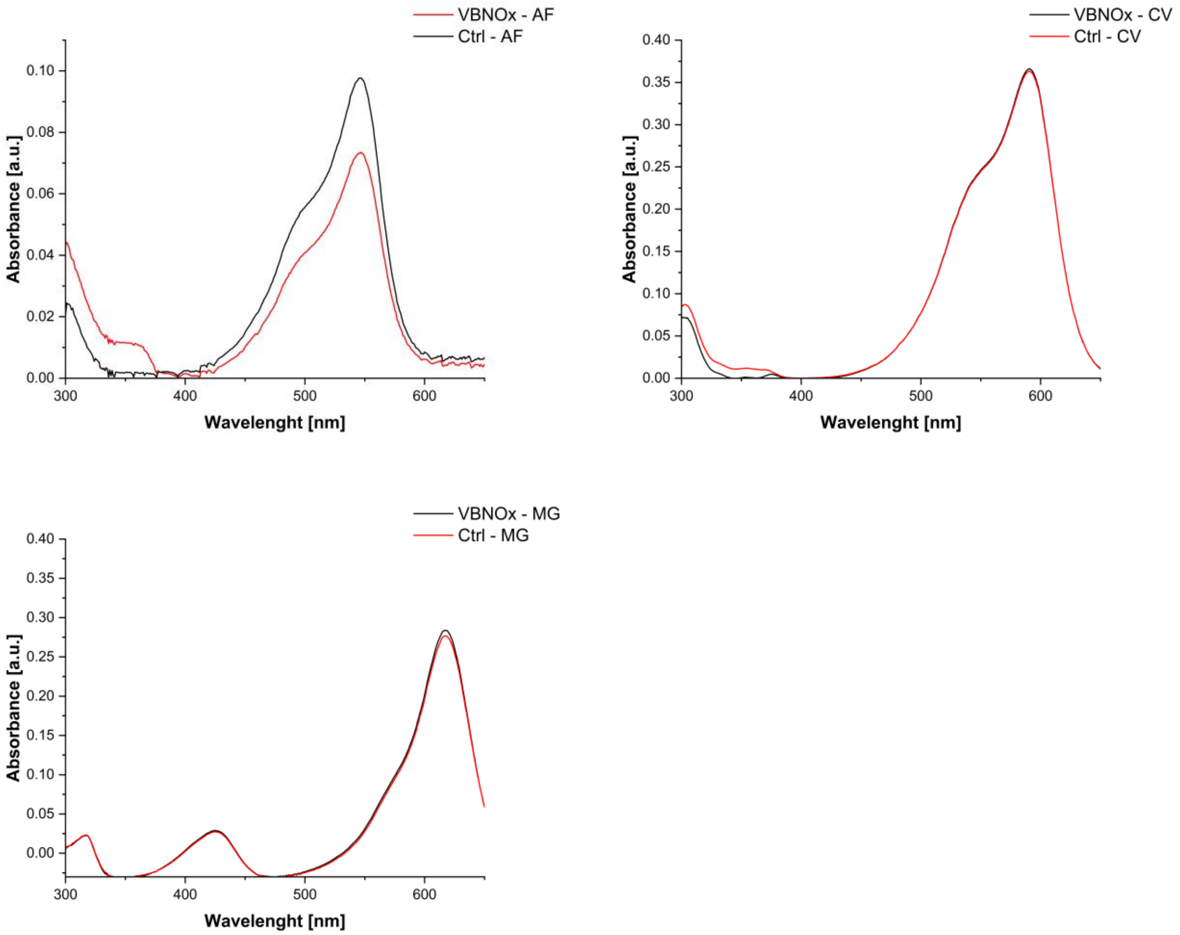

Figure S5. Fading of the color of aqueous triarylmethane dye solutions by VBNOx. A solution containing the dye (Ctrl) was used as a positive control. 10 µL of aqueous dye solution (17 mM)

were added to 5.0 mL of aqueous VBNOx solution (34 mM). The UV-Vis spectrum of the resulting solution was recorded in the range of 300-650 nm after 60 minutes of the dye adding. (A) AF. (B) CV. (C) MG.

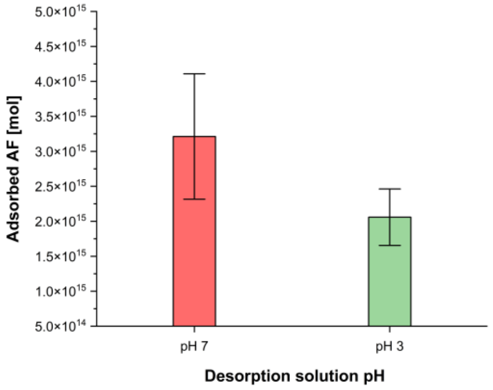

Figure S6. Effect of desorption solution pH on PE-pVBNOx. Foils were treated in 2 mL of an aqueous AF staining solution (1.0 wt%, pH adjusted to 3) for 1 h. The foils were then immersed in an aqueous washing solution at pH 3 for 10 min with ultrasonication. For desorption of the dye, the foils were treated in 5 mL of aqueous CTAC solution (1 wt%) at either pH 3 or pH 7 for 1 h at 20 °C. For desorption in CTAC at pH 7, the pH of the solution was adjusted to pH 3 with aqueous HCl solution 1 M. Results are given as mean values ± standard deviation (SD) *n* = 3.

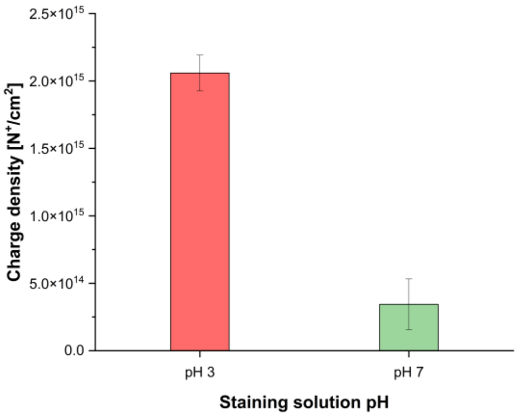

Figure S7. Effect of staining solution pH on charge density of PE-pVBNOx. Charge density values were obtained by treating the foils in 2 mL of aqueous AF solution (1 wt%, pH adjusted to 3 with aqueous HCl solution 1 M or to 7 with aqueous NaOH solution 1 M) for 1 h. The foils were then immersed in an aqueous HCl solution (pH 3) or deionized H_2O (pH 7) for 10 min with ultrasonication. For desorption of the dye, the foils were treated in 5 mL of aqueous CTAC solution (1 wt%, pH adjusted to 3 with aqueous HCl solution 1 M) for 1 h at 20 °C. Results are given as mean values ± standard deviation (SD) $n = 3$.
